# Elevated cerebral perfusion in neonatal encephalopathy is associated with neurodevelopmental impairments

**DOI:** 10.1038/s41390-024-03553-1

**Published:** 2024-09-17

**Authors:** Ruth O’Gorman Tuura, Raimund Kottke, Barbara Brotschi, Carola Sabandal, Cornelia Hagmann, Beatrice Latal

**Affiliations:** 1 Center for MR Research, University Children’s Hospital Zürich, University of Zürich (UZH), Zürich, Switzerland; 2 Children’s Research Center, University Children’s Hospital Zürich, University of Zürich (UZH), Zürich, Switzerland; 3 Department of Diagnostic Imaging, University Children’s Hospital Zürich, University of Zürich (UZH), Zürich, Switzerland; 4 Department of Neonatology and Paediatric Intensive Care, University Children’s Hospital Zürich, University of Zurich (UZH), Zürich, Switzerland; 5 Department of Anaesthesia, University Children’s Hospital Zürich, University of Zurich (UZH), Zürich, Switzerland; 6 Child Development Center, University Children’s Hospital Zurich, University of Zürich (UZH), Zürich, Switzerland

## Abstract

**Background:**

Neonatal encephalopathy (NE) represents a primary cause of neonatal death and neurodevelopmental impairments. In newborns with NE, cerebral hyperperfusion is related to an increased risk of severe adverse outcomes, but less is known about the link between perfusion and mild to moderate developmental impairments or developmental delay.

**Methods:**

Using arterial spin labelling perfusion MRI, we investigated the link between perfusion in 36 newborns with NE and developmental outcome at 2 years.

**Results:**

53% of the infants demonstrated a normal outcome at 24 months, while two had cerebral palsy with impairments in cognitive, motor, and language domains, and three infants died. The remaining infants showed mild or moderate delays in development in one or two domains. Hyperperfusion across the whole brain was associated with more adverse outcome, including an increased risk of death or severe disability such as cerebral palsy. Among the surviving infants, higher perfusion in the bilateral basal ganglia, thalamus, hippocampus and cerebellum during the neonatal period was related to a poorer cognitive outcome at 2 years.

**Conclusion:**

Hyperperfusion in infants with NE was associated with a more adverse outcome and lower cognitive outcome scores. In addition to severe adverse outcomes, altered perfusion is also related to mild to moderate impairment following HIE.

**Impact statement:**

- Neonates with neonatal encephalopathy (NE) show increased cerebral perfusion globally, which is linked to a more adverse outcome.
- Higher perfusion in the bilateral basal ganglia, thalamus, hippocampus and cerebellum during the neonatal period was related to a poorer cognitive outcome at 2 years.
- In addition to severe adverse outcomes altered perfusion is related to mild to moderate impairment following NE.
- To improve neurodevelopmental outcomes, it is important to improve our understanding of the factors influencing cerebral perfusion in infants with NE.

## Introduction

Neonatal encephalopathy (NE) is a serious neurological condition, representing one of the primary causes of neonatal death and neurodevelopmental impairment. NE is thought to affect 0.3% of live births,^1^ and is associated with a high risk of long-term severe neurological disability or mortality,^2–4^ accounting for 25% of global neonatal deaths.^5^ While therapeutic hypothermia (TH) has led to a significant decrease in neurodevelopmental morbidity after moderate and severe NE,^6–8^ there is considerable variability in outcome among infants undergoing TH, and the source of this variability remains incompletely understood. Accurate biomarkers for long-term neurodevelopmental outcome following NE are extremely important both for clinical management, outcome prediction and the evaluation of novel therapeutic approaches, aiming to improve neuroprotection and long-term outcome.

NE is associated with a cascade of biochemical changes which evolve over four distinct stages. During the initial phase of primary energy failure, the hypoxic-ischaemic insult leads to a lack of oxygen and glucose arising from a decrease in cerebral blood flow, leading to a reduction in adenosine triphosphate (ATP) and phosphocreatine, and a concomitant increase in lactate, glutamate, and reactive oxygen species.^9^ After resuscitation, a latent phase begins, during which cerebral perfusion and oxygenation are restored. This latent phase corresponds to a therapeutic window, representing an optimal time for therapeutic interventions^10^ such as TH. The latent phase is followed by a phase of secondary energy failure,^10–12^ beginning from 6 to 24 h after the hypoxic-ischaemic injury and lasting for a few days, followed by a tertiary injury phase which can last for months or years.^9^

Conventional cerebral MRI and MR spectroscopy are important markers for outcome following NE, since they are sensitive to some of the structural, microstructural, and metabolic changes which evolve with the different phases of injury.^13^ Complementary information can be obtained with perfusion MRI, which provides additional information about inflammation and cerebral vasodilation to that available from conventional MRI and MRS alone.^14^ Perfusion MRI studies of NE have revealed that perfusion is elevated from days 2 to 3 after birth,^13,15^ prior to the appearance of structural brain injury visible on MRI in the same brain regions,^15^ and exceeds the perfusion values seen in healthy newborns.^16^ Similar findings have been reported with Doppler Ultrasound, such that cerebral blood velocities within the medial cerebral artery appear to be increased in infants with severe NE at 12 h after birth.^17^ However, there has been little research into the significance of hyperperfusion for neurodevelopmental outcome, with one study showing a link between hyperperfusion and an increased risk of death or severe disability such as cerebral palsy,^18^ and another showing perfusion differences between NE infants with and without structural brain injury, which were then related to motor and language outcomes.^19^ In addition, while the patterns of brain injury accompanying moderate to severe NE have been well characterised, (for a recent review, see ref. ^13^), there is mounting evidence that infants with mild NE also demonstrate brain injury on neonatal MRI and deficits in their motor, cognitive, or language development.^13,20,21^ Since mild to moderate NE occurs more frequently than severe NE, outcome prediction is particularly relevant for this group. However, outcome prediction in infants with mild or moderate HIE remains challenging in the context of the wide spectrum of their cognitive and behavioural outcomes,^22^ underscoring the need for additional imaging biomarkers for outcome in these infants.

Arterial spin labelling (ASL) is a noninvasive MRI perfusion imaging method which uses magnetically labelled water within blood as an endogenous tracer. Labelling is typically performed with an inversion pulse, which inverts the magnetisation of water spins within blood in the feeding arteries. The labelled blood then flows into the brain and causes a transient alteration in magnetisation in the brain, which depends on the perfusion as well as the blood T1. Perfusion-weighted images can be derived by subtraction of images acquired with and without prior spin labelling, and quantitative maps of perfusion can be derived by scaling these perfusion-weighted images to a reference image and applying correction factors for a number of known physiological quantities (e.g. the T1 of blood, the blood brain partition coefficient) and technical parameters (e.g. the efficiency of the labelling pulse, the post-labelling delay between application of the label and collection of the images).^23^ The use of ASL for perfusion imaging in newborns with NE is now well established, and ASL studies have provided important insight into the evolution and timing of perfusion changes following NE.^24–26^ ASL studies have also revealed that therapeutic hypothermia does not alleviate brain injury in those newborns who show hyperperfusion on MRI, suggesting that ASL might provide a useful early marker for outcome in these infants.^13,15,16,27^

The purpose of the present study was to investigate the link between cerebral perfusion in newborns with neonatal NE, assessed with arterial spin labelling (ASL), and neurodevelopmental outcome at two years. We hypothesised that increased perfusion would be associated with lower motor, cognitive, and language outcome scores from a standardised neurodevelopmental assessment, as well as with impairments or developmental delays in multiple neurodevelopmental domains.

## Materials and methods

### Participant group

The participant group consisted of 37 term-born neonates, (see Table 1 for demographics) all of whom were outborn between 2011 and 2019. On admission to the paediatric intensive care unit, the neonates were clinically examined by a senior consultant and staged according to the Sarnat score.^28^ Neonates eligible for hypothermia (*N* = 34) were cooled to a core temperature of 33.5 degrees (range 33–34) for 72 h according to Swiss hypothermia guidelines.^29^ MRI was performed at a mean age of five days, and the minimum time between rewarming and MRI was 12 h. The study was approved by the cantonal ethics committee of the canton of Zürich, Switzerland, and all parents gave written informed consent for participation.

**Table 1:** Participant demographics and clinical characteristics

Variables	Total *N* 36
Female participants, *N* (%)	20 (56%)
Treated with hypothermia, *N* (%)	34 (94%)
Sarnat 1, *N* (%)^a^	1^a^
Sarnat 2, *N* (%)	24
Sarnat 3, *N* (%)	11
Age at MRI, M (SD) (days)	5.1 (1.3)
Rutherford score^30^	3.4 (2.6)
Weeke score^31^	12 (11)
Trivedi score^32^	24 (29)
Age at follow-up, M (SD) (months)	24 (2.6)
Bayley cognitive composite score (CCS) at follow-up	102 (18)
Bayley language composite score (LCS) at follow-up	95 (21)
Bayley motor composite score (MCS) at follow-up	96 (20
Outcome: 0 (no delay/impairment at follow-up), *N* (%)	19 (53%)
Outcome: 1 (delay/impairment in 1 domain), *N* (%)	6 (17%)
Outcome: 2 (delay/impairment in 2 domains), *N* (%)	6 (17%)
Outcome: 3 (delay/impairment in 3 domains), *N* (%)	2 (6%)
Outcome: 4 (death), *N* (%)	3 (8%)

^a^In accordance with the therapeutic hypothermia protocol, the infant with a Sarnat score of 1 was not treated with hypothermia.

### MRI data collection

Conventional MRI data included sagittal, axial, and coronal T2-weighted fast spin echo (FSE) images, T1-weighted spin echo and 3D spoiled gradient echo images, axial diffusion tensor imaging, and single voxel spectroscopy of the basal ganglia and white matter. For all conventional sequences the in-plane resolution varied from 0.3 to 0.7 mm with a slice thickness of 1–3 mm. Injury scores were derived from the conventional MRI data following the scoring systems from Rutherford et al.^30^ Weeke et al.^31^ and Trivedi et al.^32^

ASL perfusion MRI data were collected with a background-suppressed, pseudocontinuous ASL sequence with a 3D stack of spirals readout, with a field of view of 24 cm (*n* = 11) or 18 cm (*n* = 26), a matrix of 128 × 128 and a slice thickness of 4 mm. 28 infants were scanned with a 3 T GE MR750 scanner, while the remaining infants were scanned with 3 T GE HD.xt (*n* = 8) or 1.5 T GE MR450 (*n* = 1) scanners. The subgroup of infants scanned on the 3 T GE HD.xt scanner included proportionally more infants with a Sarnat 3, and the one infant scanned at 1.5 T also had a Sarnat score of 3. A post-labelling delay of 2 seconds was used at 3 T and 1.5 seconds was used at 1.5 T. Perfusion was quantified using the default model implemented in the online perfusion reconstruction provided by the scanner vendor,^23^ accounting for differences in tissue and blood T1 between 1.5 T and 3 T. Perfusion maps were normalised to a neonatal perfusion template using FSL-FLIRT,^33^ and the average whole-brain perfusion and basal ganglia perfusion were extracted for each participant, using the basal ganglia and grey matter masks derived from the automated anatomical labelling (AAL) atlas, after normalisation to the neonatal template.

### Neurodevelopmental assessment

Neurodevelopmental follow-up data were collected at a mean age of 24 ± 2.6 months (Mean ± SD). Participants were evaluated by an experienced paediatric neurologist or developmental paediatrician, using the Bayley Scales of Infant and Toddler Development, 3^rd^ edition (Bayley-III) (*n* = 35),^34^ the Bayley-II (*n* = 1),^35^ or the Griffiths neurodevelopmental assessment (*n* = 1).^36^ Outcome was categorised as: 0 (no impairment observed), 1 (impairment in one developmental domain), 2 (impairment in two domains), 3 (impairment in 3 domains), or 4 (death). Impairment for a particular domain was defined as a composite score of less than 85 for the Bayley-III for the specific domains (cognitive composite score (CCS), language composite score (LCS), or motor composite score (MCS)).^37^ The patient examined with the Griffiths neurodevelopmental assessment demonstrated normal development in all domains at follow-up, so this patient was assigned to the normal outcome group (group 0), but was excluded from analyses of individual domains. For the patient assessed with the Bayley-II, the mental developmental index was converted to a Bayley-III cognitive composite score.^38^ Permutation testing was performed to test for statistically significant correlations between the voxelwise perfusion and neurodevelopmental outcome scores (ranging from 0 to 4 as described above) or the individual Bayley-III domains.

### Statistical analyses

Potential effects of the scanner used for data acquisition on the measured perfusion values were assessed with a Kruskal–Wallis test, and the association between age at MRI and whole-brain perfusion was tested using a bivariate Spearman correlation after confirming non-normality of the age and scanner index data with a Shapiro–Wilk test. Additional correlations between the average whole brain perfusion and outcome scores were performed both with and without correction of the blood T1 value for the haematocrit.^39^ The sensitivity and specificity of the basal ganglia perfusion values and brain injury scores to outcome were assessed with a receiver operating curve (ROC) analysis, including a predictor variable from a binary logistic regression model including both the basal ganglia perfusion and the brain injury score as regressors in the model. The ROC analyses were performed to assess the sensitivity and specificity both to severe adverse outcome (defined a death or presence of cerebral palsy, for consistency with previous studies^18^), and for any developmental impairment at follow-up (outcome score greater than or equal to 1). Differences of risk factors between outcome groups were analysed with the Kruskal–Wallis test. Statistical analyses were performed with SPSS version 27. Voxelwise perfusion was compared to the neurodevelopmental outcome using FSL-randomise (FMRIB software library: https://fsl.fmrib.ox.ac.uk/fsl/).^40^ To account for any potential confounds arising from the differences between scanners used for data collection, a covariate accounting for the scanner on which the data were acquired was used in all correlation analyses between perfusion and outcome. The significance threshold was set to *p* < 0.05, correcting for multiple comparisons with threshold free cluster enhancement (TFCE).^41^

## Results

One infant with myotonic dystrophy was excluded from the analysis, resulting in a group of 36 infants, of whom 19 (53%) demonstrated a normal outcome at 24 months, while six showed a mild impairment in language development (Bayley-III language composite scores of 78–84), but normal cognitive and motor development. Five infants showed impairment in two domains, two had cerebral palsy with impairments in all three domains, and three infants died (Table 1). The two patients with cerebral palsy had dystonic, bilateral-spastic cerebral palsy (GMCFS III) and dyskinetic cerebral palsy (GMCFS II), respectively.

The distributions of brain injury scores are shown in Table 1 and supplemental Fig. 1. The majority of infants had brain injury scores falling in the lower half of the range for each score, suggesting a mostly mild to moderate profile of brain injuries across the group, although the observed severity spanned the full range of scores for each scale. For the Rutherford score, 6 infants demonstrated normal appearances on conventional MRI, while only two infants had a normal score of 0 according to the Weeke score, although this score also includes some findings unrelated to NE such as subdural haematoma and sinus thrombosis. For the Trivedi score, 7 infants demonstrated normal appearances on conventional MRI (cumulative score = 0), 12 infants were classified as having mild injury (cumulative score: 1–11), 11 infants showed moderate injury (cumulative score: 12–32), and 6 infants showed severe injury (cumulative score ≥33). The predictive accuracy, (quantified as the area under the ROC curve) for the Rutherford, Trivedi, and Weeke scores was (0.74, 0.74, 0.75) for any impairment, and 0.91, 0.97, and 0.94 for severe adverse outcome, respectively. The Trivedi score demonstrated slightly higher predictive accuracy for severe adverse outcome, while all three scores demonstrated comparable predictive accuracy for any impairment.

No significant effects of post-natal age on perfusion were observed, but a significant confounding effect of the scanner was observed (*p* = 0.004), possibly due to differences in the severity of brain injury across scanners. The injury scores were also higher for the newborns scanned on the 3 T HD.xt scanner, although this difference did not reach significance for any of the scores (all *p* > 0.12).

**Fig. 1:**
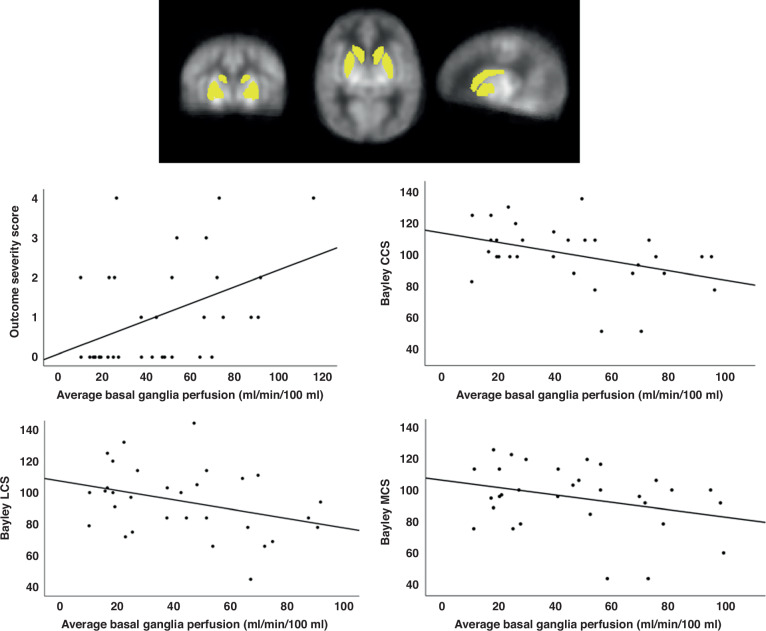
Scatter plots showing the association between the basal ganglia perfusion values extracted using the automatic anatomic labelling (AAL) atlas, the outcome severity score, and the Bayley CCS, LCS, and MCS. The average perfusion map from the group is shown in the top panel,  with the basal ganglia mask from the AAL template superimposed. Lower panels depict the association between the basal ganglia perfusion and the outcome severity score (Spearman’s rho = 0.475, *p* = 0.004), and between the basal ganglia perfusion and the Bayley cognitive composite scores (CCS, Spearman’s rho = −0.435, *p* = 0.015), language composite scores (LCS, Spearman’s rho = −0.351, *p* = 0.053) and motor composite scores (MCS, Spearman’s rho = −0.223, *p* = 0.227).

Correlations between the average basal ganglia perfusion and outcome severity score ranging from 0 (normal outcome) to 4 (death) revealed a significant positive association (Spearman’s rho = 0.475, *p* = 0.004, Fig. 1), which remained significant after excluding the 4 patients who died and the 2 patients who developed cerebral palsy (Spearman’s rho = 0.374, *p* = 0.041). Correlations between the basal ganglia perfusion and the Bayley-III composite scores revealed a significant negative correlation between the basal ganglia perfusion and CCS (Spearman’s rho = −0.435, *p* = 0.015), a trend-level negative association between basal ganglia perfusion and LCS (Spearman’s rho = −0.351, *p* = 0.053), and no association between basal ganglia perfusion and MCS (Spearman’s rho = −0.223, *p* = 0.227).

**Fig. 2:**
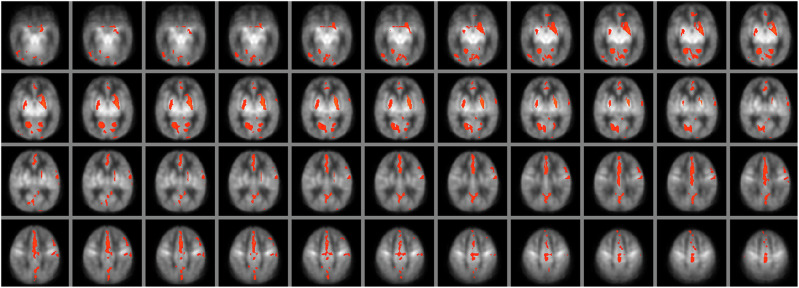
Voxelwise correlation analysis between perfusion and outcome. Significant clusters are overlaid in red, depicting areas in which the perfusion is positively correlated with outcome, ranging from 0 (no impairment) to 4 (severe adverse outcome). Clusters are overlaid on the HIE perfusion template. Significant clusters are seen in the basal ganglia and thalamus, as well as in a diffuse set of cortical regions, indicative of a global association between perfusion and outcome (*p* < 0.05, corrected).

**Fig. 3:**
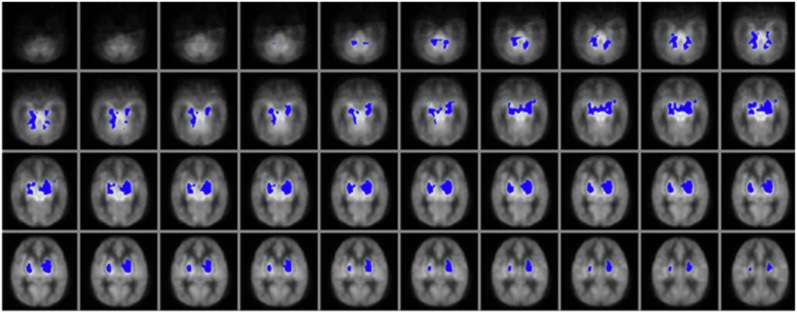
Voxelwise correlation analysis between perfusion and the Bayley-III cognitive composite score (CCS). Significant clusters are overlaid in blue, depicting areas in which the perfusion is negatively correlated with CCS (where higher scores indicate a better cognitive outcome). Significant clusters are seen in the bilateral basal ganglia and thalamus, extending into the hippocampus and anterior cerebellum (*p* < 0.05, corrected).

In the voxelwise correlation analyses, increased perfusion was positively associated with a more adverse outcome, consistent with the findings observed for the basal ganglia perfusion values extracted with the AAL atlas. This association was observed bilaterally in the basal ganglia and a diffuse set of cortical regions, suggestive of a global rather than regional association between perfusion and outcome (Fig. 2). Voxelwise correlation analyses between perfusion and the individual Bayley domains revealed a significant negative association between cognitive outcome and perfusion in the bilateral basal ganglia, with clusters extending into the hippocampus and the anterior part of the superior cerebellum (Fig. 3). This relationship remained significant and even became more widespread after excluding the two patients with cerebral palsy from the analysis (Supplementary Fig. 2). In summary, higher perfusion was therefore associated with a higher (more adverse) outcome severity score, and lower Bayley cognitive composite scores. No significant correlations were observed between perfusion and motor or language outcome, after correction for multiple comparisons with TFCE. No significant differences in haematocrit were observed between outcome groups (*p* = 0.485), and the correlation between whole brain perfusion and outcome remained comparable after correction of the perfusion values for blood T1 differences arising from the inter-individual differences in haematocrit (without haematocrit correction: Rho = 0.401, *p* = 0.015; with haematocrit correction: rho = 0.352, *p* = 0.035).

The sensitivity and specificity of the basal ganglia perfusion values to severe adverse outcome and to any impairment at follow-up are given in Tables 2 and 3, respectively. For the predictive accuracy to severe adverse outcome, the brain injury score from Trivedi et al.^32^ provided the highest combined sensitivity and specificity, but for the predictive accuracy to any impairment at follow-up, the perfusion offered comparable sensitivity and higher specificity to outcome than the brain injury score from conventional MRI alone (Table 3). However, the highest predictive accuracy to any impairment at follow-up was associated with the predictor variable from the binary logistic regression combining both the basal ganglia perfusion and the brain injury score into a single variable. The binary logistic regression model was statistically significant (χ2(2) = 16.943, *p* < 0.001) and explained 51% (Nagelkerke R2) of the variance in outcome (any impairment at follow-up). In the model, both the average basal ganglia perfusion and the Trivedi score were statistically significant (BG perfusion: B = 0.056, Wald = 7.031, *p* = 0.008; Trivedi score: B = 0.049, Wald = 4.927, *p* = 0.026).

**Table 2:** Sensitivity and specificity of perfusion and brain injury on conventional MRI to severe adverse outcome (Outcome score = 4)

	AUC	Sensitivity	Specificity
Basal ganglia (BG) perfusion >52 ml/min/100 ml	0.77	80%	73%
Trivedi score >30	0.97	100%	87%
Predictor variable (BG perfusion & brain injury)	0.96	100%	80%

**Table 3:** Sensitivity and specificity of perfusion and brain injury on conventional MRI to any impairment (Outcome score >0)

	AUC	Sensitivity	Specificity
Basal ganglia perfusion >50 ml/min/100 ml	0.80	65%	83%
Trivedi score >17	0.76	65%	72%
Predictor variable (BG perfusion & brain injury)	0.86	88%	78%

## Discussion

The link between basal ganglia perfusion and severe adverse outcome from NE has been described previously.^18,27^ However, the present study extends these findings by demonstrating firstly that the correlation between perfusion and adverse outcome is also present for cortical regions and may therefore represent a global effect, and secondly, that elevated perfusion is also linked to mild or moderate impairment as well as severe adverse outcomes. The latter observation is of high clinical relevance, given the broad spectrum of outcomes observed, particularly in infants with moderate NE, and the need for imaging biomarkers for outcome in this population. In addition, the correlation analyses with the cognitive composite scores revealed an association between cognitive outcomes and regional perfusion in the basal ganglia, hippocampus, and anterior cerebellum. To our knowledge, this association has not been described previously, and underscores the importance of subcortical and cerebellar regions for cognitive as well as motor development during the neonatal period.

Unlike results from another recent NE perfusion study,^19^ we did not observe a link between perfusion and motor or language outcome. The lack of a significant association with language outcome is surprising in light of the low language scores in the group with mild impairment, but may be due to a lack of statistical power, considering the smaller effect sizes for the association between LCS and perfusion, in comparison to that between CCS and perfusion. In general, the correlation analyses with the individual Bayley domains may be underpowered in comparison to that for the outcome summary scores, since the Bayley data were only collected from the group of surviving infants, in whom only a small subset demonstrated adverse outcomes. The correlation therefore depends heavily on the data from the few infants with lower CCS scores, and should be replicated in a larger sample. However, the link between regional perfusion in the basal ganglia, hippocampus and cerebellum and cognitive outcome in HIE infants is interesting in light of the known association between reduced basal ganglia, hippocampal and cerebellar volumes and cognitive outcome in other groups of high-risk infants, such as those born preterm or with congenital heart disease.^42–45^ Reduced hippocampal volumes and altered cerebellar microstructure have also been reported previously in HIE.^46,47^ These structures are particularly vulnerable to hypoxic injury during the neonatal period, and we can speculate that the hemodynamic changes may precede a subsequent loss of volume, as seen in the case of stroke, diaschisis, or neurodegenerative changes.^48–50^ Perfusion changes in these vulnerable structures may therefore represent early markers for brain injury and volume loss, but future studies with serial MRI would be needed to elucidate the link between hyperperfusion, subsequent volume loss and outcome.

In previous studies investigating the sensitivity and specificity of ASL perfusion values to outcome in infants with HIE, cut off values for basal ganglia and thalamus perfusion were reported to range from 28.75 ml/100 g/min^51^ to 51 ml/100 g/ml,^18^ for predicting adverse outcomes. The range of cut off values reported might be due to differences in the ASL methods used for data collection, but could also be due to the difference in outcome measures, quantified in one study as death or severe disability by 18 months,^18^ and in another study as a score >35 on the Neonatal Behavioural Neurological Assessment at one month.^51^ These previous studies reported sensitivities ranging from 86 to 92%, while the specificity with the respective cut offs was 100% for both studies.^18,51^ In the present study, the optimal perfusion threshold for distinguishing positive from adverse outcomes was a basal ganglia perfusion of 50 ml/100 g/min, which provided 80% sensitivity to severe adverse outcome and 65% sensitivity to any impairment (defined as a Bayley composite score <85 in any of the cognitive, motor, or language domains), and 73% specificity to severe adverse outcome and 83% specificity to any impairment. The corresponding AUC values (0.77 for severe disability, 0.80 for any impairment), were also lower than those reported previously (ranging from 0.92 to 0.99). This discrepancy may be due in part to the timing of the scan, since elevated perfusion present in neonates with HIE during the first three days after birth has been reported to decrease between 3 and 7 days after birth,^26,52^ and the infants in the present study were scanned predominantly during this time, with a mean age at scan of 5 days. However, the range of ages at MRI for the infants in the present study is comparable to that reported previously (mean 4.5 days, range 2–7)^18^ vs mean 5 days, range 3–9 days in the present study. Alternatively, differences in the predictive accuracy of the basal ganglia perfusion for outcome may reflect differences in the severity of brain injury between study cohorts. In the present sample, the majority of the infants (67%) had a Sarnat score of 2, associated with moderate HIE, and the brain injury scores showed a higher proportion of infants in the lower range of each score (Supplementary Fig. 1), reflecting a mostly mild to moderate profile of brain injuries across the group, despite the under-representation of Sarnat-1 infants within the sample. In contrast, previous studies included cohorts with a higher proportion of severely affected infants, in whom changes are more pronounced and adverse outcomes are more common, (for example, the mortality rate reported in the study by De Vis et al. was 21%,^18^ in comparison to 8% in the present study). The modest predictive accuracy of basal ganglia perfusion values from the present cohort therefore underscores the challenges of accurate outcome prediction in infants with moderate HIE, but the lower specificity also has high clinical relevance, indicating that hyperperfusion during the neonatal period does not always lead to an adverse outcome. This finding is important for clinical management and for counselling of parents, although future studies with larger sample sizes and including a larger group of Sarnat 1 infants would be needed to clarify and extend these findings

In addition, the predictive accuracy to any impairment including mild developmental delay was improved by combining perfusion data with injury scores from structural MRI, although for the severe adverse outcome (death or cerebral palsy), the best predictive accuracy was from the injury scores from conventional MRI alone. The significance of the regressors both for perfusion and for brain injury in the binary logistic regression model indicates that these measures provide complementary information for characterising the brain changes leading to any impairment at follow-up, but one important limitation of this work is that both the perfusion images and the follow-up assessments were only acquired at a single time point. The hyperperfusion following HIE is thought to be driven by the severity of brain injury during the secondary phase of brain injury (12–72 h after the initial hypoxic-ischaemic event). Therefore, the time point of maximally increased perfusion depends on the exact timing of the initial injury, which is often not known. In future, serial ASL studies may be able to characterise the trajectory of altered perfusion, and provide a more accurate assessment of the maximally increased perfusion, although repeated MRI scans in NE are challenging, particularly when the infants are clinically unstable. Future assessments of longer-term outcome would also be needed to clarify the presence and nature of developmental impairments arising later in childhood, and follow-up assessments at multiple time points would help to clarify if the observed changes represent impairments or developmental delays, with a potential catch-up or progression over time.

Another important limitation to consider is the use of multiple scanners for data acquisition. The significant effect of the scanner used for data acquisition on the whole-brain perfusion may reflect differences in injury severity, since the subgroup of infants scanned on the HD.xt scanner included proportionally more Sarnat 3 infants, and only one infant (also with a Sarnat score of 3) was scanned at 1.5 T. However, differences in estimated perfusion values between scanners of differing field strengths could also arise from differences MR sequence-related factors like the efficiency of the background suppression, which is typically optimised to a range of grey-matter T1 values. Since the T1 relaxation times are longer at 3 T than at 1.5 T, and are longer in neonates than in older children or adults, the grey matter T1 values in neonates scanned at 3 T may exceed the range of T1 values for which the background suppression is optimised. Technical factors like the efficiency of the inversion pulse or the background suppression should therefore be investigated further in a larger sample of neonates, to confirm the generalisability of results before perfusion values are applied in predictive models of outcome using a fixed perfusion threshold. Alternatively, the efficiency could be estimated in vivo in each case by scaling the average whole brain perfusion from ASL to that from the arterial inflow from phase contrast MR angiography divided by the total brain volume.^53^

The higher haematocrit seen in newborns in comparison to that in older children and adults is associated with lower T1 values,^39^ and the assumption of a standard blood T1 value (of 1650 ms at 3 T and 1350 ms at 1.5 T^23^) in the model used for perfusion quantification may lead to a systematic underestimate of the perfusion. However, in the present study correction for the haematocrit did not alter the association between perfusion and outcome, and the haematocrit did not differ between outcome groups. The apparent underestimate of perfusion arising from the higher neonatal haematocrit therefore appears to be a systematic effect which does not alter associations with outcome within a single site, but should be taken into account when comparing perfusion values between sites or scanners, depending on the model used for quantification and whether or not the haematocrit was used to derive an individual blood T1 value. An additional potential source of error in the quantification of perfusion in neonates with HIE is the brain temperature, since the blood T1 also varies with temperature.^39^

## Conclusion

In a cohort of NE infants with predominantly moderate NE, undergoing therapeutic hypothermia, globally elevated perfusion was associated with an adverse outcome, and a lower perfusion in the basal ganglia, thalamus, hippocampus, and cerebellum was linked to a better cognitive outcome. However, other neuroimaging markers should be considered in combination with ASL data for prediction of outcome following NE.

## Supplementary information

Supporting Information

## Data Availability

The datasets generated during and/or analysed during the current study are available from the corresponding author on reasonable request, subject to the required ethical approval for data sharing.
