# Supplementary material for: Elevated cerebral perfusion in neonatal encephalopathy is associated with neurodevelopmental impairments
Source: Pediatr Res. 2024 Sep 17;97(5):1597–604. doi: 10.1038/s41390-024-03553-1 (PMC12119360; doi:10.1038/s41390-024-03553-1)
Supplement: Supplementary file 1 — Supporting Information [file 41390_2024_3553_MOESM1_ESM.pdf]

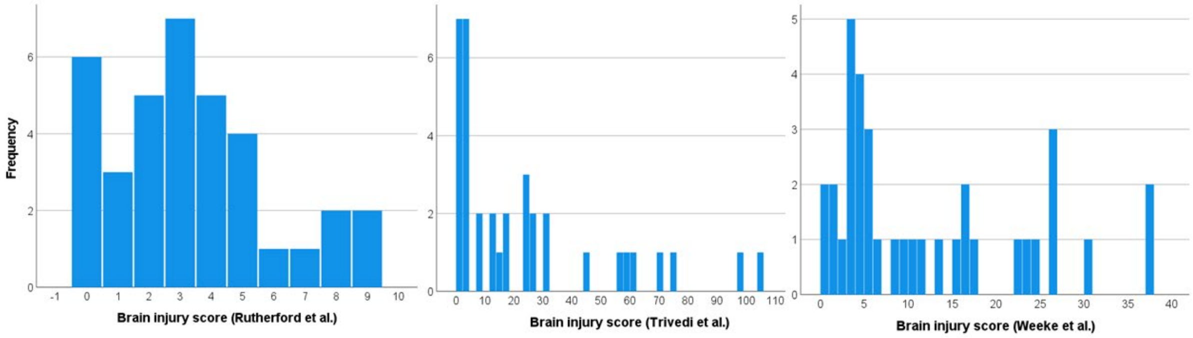

Supplemental figure S1: Distribution of brain injury scores, calculated from the structural MRI data according to the Rutherford, Trivedi, and Weeke scores, respectively.

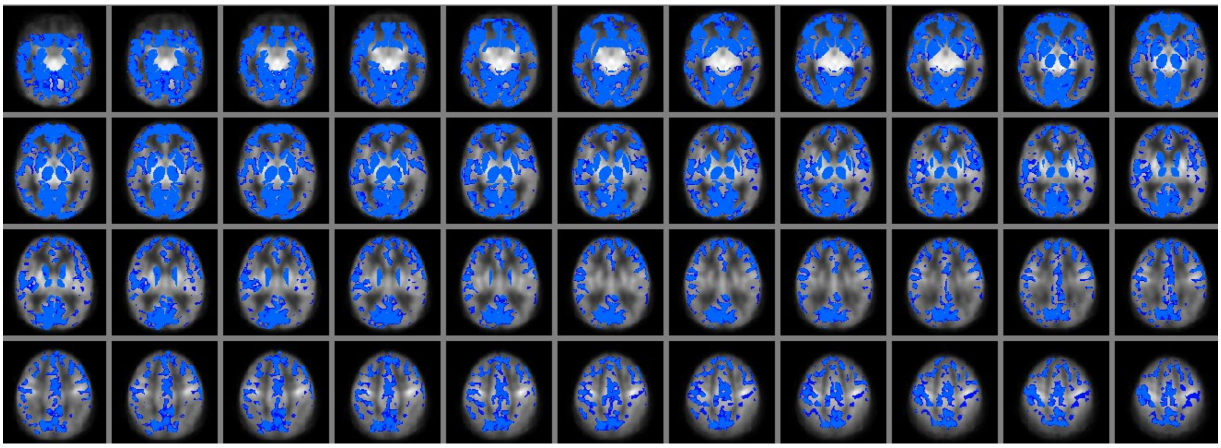

Supplemental figure S2: Voxelwise correlation analysis between perfusion and the Bayley-III cognitive composite score (CCS), after excluding the two patients who developed cerebral palsy. Significant clusters are overlaid in blue, depicting areas in which the perfusion is negatively correlated with CCS (where higher scores indicate a better cognitive outcome).
